# “Float[ing] in the Middle” Nurse Navigators and the Interface of Access to Care

**DOI:** 10.3390/ijerph22111631

**Published:** 2025-10-26

**Authors:** Clare Hannan-Jones, Lisa Fitzgerald, Geoffrey Mitchell, Allyson Mutch

**Affiliations:** Faculty of Health, Medicine, and Behavioural Sciences, School of Public Health, The University of Queensland, Herston, QLD 4006, Australia

**Keywords:** nurse navigator, complex care, access to health care, person-centred care, integrated care, patient navigation, health and social care

## Abstract

The Australian health care system continues to struggle to meet the needs of people experiencing multiple complex chronic conditions. Australians who report poorer health continue to report poorer access to health care. Inequities in access are attributed to a “mistmatch” between the health care system and individuals’ clinical and social needs. To address this misalignment at the interface of access, innovative approaches that consider both individual and system-level barriers to care need to be examined. Nurse navigation models designed to support people negotiating complex care and bridge systems and service gaps have been touted as a method to enhance access, but how nurse navigators work at the interface of access in practice is unclear. This qualitative study examined the mechanisms by which nurse navigators facilitate access to care for people experiencing complex care needs through an exploration of key stakeholder perspectives: nurse navigators, nurse navigator patients, and care professionals. Data collection involved in-depth semi-structured interviews, and analysis included reflexive thematic analysis and data triangulation processes. A conceptual framework of access to health care was used to explore nurse navigators’ roles at both system and patient levels. Nurse navigators supported both patients and care professionals by building relationships across the interface of access, challenging norms of care, and facilitating empowerment. Nurse navigators acted as intermediaries to negotiate access, work made possible through their knowledge of systems and capacity to identify and respond to multidimensional care needs and systems challenges. This research highlights the importance of holistic and relational approaches to overcome issues of access for all involved.

## 1. Introduction

Persistent inequities in access to health care are attributed to a misalignment between health systems and people’s complex care needs [1]. To improve health care access, particularly for people with complex needs, health system- and individual-level challenges must be addressed [2,3]. Integrated and person-centred approaches are touted as best practice for addressing barriers to health care access, but these aspirations can get “lost in translation”, conflicting with disease-centric health care structures and practices [4,5,6]. Instead, complex and fragmented health systems continue to prioritise system needs and deflect problems of access to individuals (e.g., an individual’s lack of knowledge of service availability or eligibility is attributed to the patient, not the system) [7]. Practical strategies that challenge barriers to access by engaging meaningfully at the interface of health systems and people are needed to address these issues. This study considers how nurse navigation, a novel strategy designed to address barriers to access for people with complex health and social needs, operates at this interface.

### 1.1. Understanding Access

In 2013, Levesque et al. [3] developed a conceptual framework to map access across both health system dimensions (on the supply side) and individuals’ abilities (on the demand side). They defined access as a multidimensional process that incorporates more than financial or geographical access, but that spans from perceiving a need for care, seeking, reaching, and using care to having a beneficial outcome from the care received. Along this multidimensional process, the authors identified five independent but interacting service level dimensions and five corresponding abilities of individuals that mediate access [3]. Importantly, the interface of these dimensions and abilities is not unidirectional but consists of dynamic interconnections between individuals and systems that are bidirectional and in constant negotiation across the process of access [3]. As a consequence, the interface of access functions at both discrete points and across the process of access.

Levesque et al. [3] provided a detailed consideration of access but also acknowledged limitations of their framework. They called on researchers to explore access across different contexts, to deepen understanding of the interface between system dimensions and people’s abilities. To date, researchers have taken up this challenge, suggesting many adaptations [2,8,9,10,11]. For example, Davey et al. [12] examined the applicability of Levesque et al.’s framework in Indigenous health primary care settings and recommended that the interrelatedness of dimensions and abilities be better emphasised through non-linear representations of the framework [12]. Others have called for greater acknowledgement of the influence of policy and structural system features on determining access from the supply side [11,12,13]. Voorhees et al. [13] and Song et al. [14] highlighted the need to capture lay experiences of access, acknowledging that the health workforce also shapes experiences of access to care. Richard et al. [2] further argued that research is needed to explore what happens at the interface itself, to clarify how “supply” and “demand” relate to each other in practice. Hence, we need to consider how understandings of access, as multidimensional relationships between diverse stakeholders interacting at the interface of individuals and the health system, operate in practice. In this study, we consider nurse navigation as a model of care operating at this interface to address barriers to health care access for people with highly complex care needs.

### 1.2. Nurse Navigation

Patient navigation emerged in the 1990s in the United States as a method to remove barriers to timely care for marginalised women accessing cancer screening and care [15]. Since then, key model principles have been widely accepted, including person-centred integrated care, the elimination of barriers to care, operation across health and social systems, and facilitation of timely transitions to appropriate care [15,16]. Patient navigation models can engage a range of professionals (e.g., social workers, mental health workers, nurses) or lay people in the navigation role, but a model receiving increasing attention engages nurse navigators (NNs) to strengthen patient access while also fostering health system adaptions that may enhance access (e.g., streamlining communication processes between hospital departments) [17,18,19].

In Australia, nurse navigation models are typically designed to negotiate barriers to care for people experiencing complex health and social needs [20,21,22,23]. NNs respond to both the supply and demand sides of access, working to support patients while also seeking to adapt services and systems [17,18,19,22,23,24,25,26,27,28]. However, given the variability and complexity of people’s care needs, how NNs negotiate access to health care in practice, working with individuals and other care professionals, remains unclear. Further research is needed to explore the operationalisation of NN models in practice, particularly in relation to health care access [22,23,26,27,28].

This research explores how nurse navigation operates at the interface between individuals and health systems to facilitate access to health care. Specifically, this study draws on the experiences of three key stakeholder groups, NNs, NN patients, and care professionals, to examine the inner workings of NN practice. This in-depth exploration of stakeholders’ experiences will examine how NNs address the (many) disconnects between individuals’ abilities and health system dimensions of access to health care.

## 2. Methods

**Figure A1:**
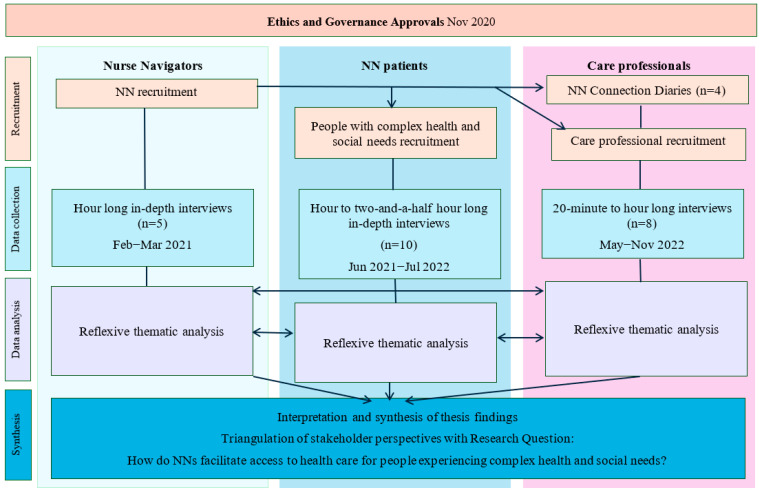
Research design overview.

A multistage, qualitative study was designed to investigate NNs’ role in facilitating access to care for people with complex needs (see Figure A1) [29]. Key stakeholder perspectives were explored via in-depth interviews to give multidimensional insight into experiences of access to health care facilitated through NNs.

This study focused on the nurse navigation service operating within the West Moreton Health and Hospital Service (WMHHS), a large health district situated in southeast Queensland, Australia. This sociodemographically unique region includes a geographically dispersed and ageing population living in regional and rural areas [30]. The area has seen exponential population growth in recent years, with many experiencing persistent social disadvantages and health inequalities. The region’s health profile includes higher than state average rates of chronic disease, obesity, premature death, smoking, and potentially preventable hospitalisations, all of which are putting increased demands on existing services [31]. The nurse navigation service supports people experiencing multiple chronic conditions and frailty. It was designed to respond to their health and social needs and improve health and service outcomes [31]. The WMHHS model has NNs reporting directly to the Director of Nursing, which allows them to work across disciplinary and departmental boundaries. This study was designed in collaboration with the Director of Nursing and Midwifery and the NN team. Ongoing consultation ensured the study was of value to the NN team and the research was acceptable, appropriate, and practically achievable [32].

### 2.1. Participant Recruitment and Data Collection

Participant recruitment and data collection were systematically undertaken for the three stakeholder groups—NNs, NN patients, and care professionals. The following provides a description of recruitment and data collection for the three stakeholder groups. The consolidated criteria for reporting qualitative research checklist was used to guide this description [33].

**Nurse Navigators.** At the time of data collection, 16 NNs were employed by WMHHS across various specialities, including palliative care, aged care, diabetes transitions, adult disability, complex surgical transitions, and emergency department frequent admissions. All NNs employed by WMHHS received a presentation from the research team about the study. Prospective NN participants were invited to participate in an hour-long interview, distribute study information to patients, and complete a connection diary to document the types of care professionals they worked with. The final sample of five NN participants from different specialties was identified in consultation with the NN team to represent the breadth of practice. The participating NNs’ specialties spanned chronic disease, key population groups, and/or specific hospital settings. All had extensive experience in their area and had been employed as an NN for between 18 months and five years.

Five NN interviews were conducted face-to-face in a clinical setting by two researchers (CHJ and AM) between February and March 2021 and lasted for an hour. Interviews followed a semi-structured format, broadly structured around Levesque et al.’s [3] framework, with questions designed to explore NN roles and activities negotiating patient abilities and health system dimensions of access (see Document S1).

**NN Patients.** To be eligible, participants needed to be patients currently or previously engaged with a WMHHS NN. Seven NNs (the five interviewees and two additional NNs) volunteered to distribute invitation flyers and contact forms to patients, following convenience sampling techniques. The contact form outlined that expressions of interest would not affect current or future care, and contact information would be kept confidential and only used for the purpose of the research team providing study information. CHJ then followed up via phone and email. Between June 2021 to July 2022, ten NN patients were recruited. Participants were a heterogeneous sample from diverse backgrounds and ages, with intersecting health and social needs.

Ten in-depth interviews with NN patients were completed—eight were conducted by two researchers (CHJ and AM) and two by CHJ between June 2021 and July 2022. Two interviews also involved a caregiver. Interviews went for between 45 min and two and a half hours. All interviews were conducted face-to-face in the participants’ homes. Interviews examined people’s experiences of access to health care across the life course and the NN’s role in facilitating access (see Document S2).

**Care Professionals.** Connection diaries were developed by the research team in consultation with the NN team to record the types of professionals with whom NNs engaged and to identify potential care professional participants (see Document S4). Four of the NNs who participated in an interview consented to complete connection diaries. Returned diaries illustrated the diversity of care professionals that NNs connected with across community-based social care settings (e.g., housing support services, local charities, and welfare services) and primary, secondary, and tertiary health care settings.

Information gathered through the connection diaries was used to identify participants for interviews. To be eligible, participants needed to have some form of professional interaction with an NN. From March 2022 to November 2022, the research team purposefully sampled general practitioners (GPs), allied health professionals (AHPs), and community-based social and welfare professionals (hereafter collectively referred to as community-based social professionals (CSPs)) identified through the connection diaries. This purposive sampling strategy was intended to capture the heterogeneity of NN engagement with care professionals. Prospective participants were approached via email, letters, and phone. The final sample of eight participants included professionals who operated in community, primary, and secondary health care settings and included GPs, a nurse practitioner, AHPs, and CSPs.

Eight interviews were completed between May and November 2022. Interviews were conducted by CHJ face to face in workplaces and via phone or video conferencing software. They ranged from 20 min to one hour in length. Interviews focused on participants’ experiences supporting people with complex needs and their work with NNs (see Document S3). Field notes were compiled at the completion of each interview. All interviews were transcribed verbatim, deidentified, and imported into NVivo 12 software for analysis.

### 2.2. Data Analysis

**Figure A2:**
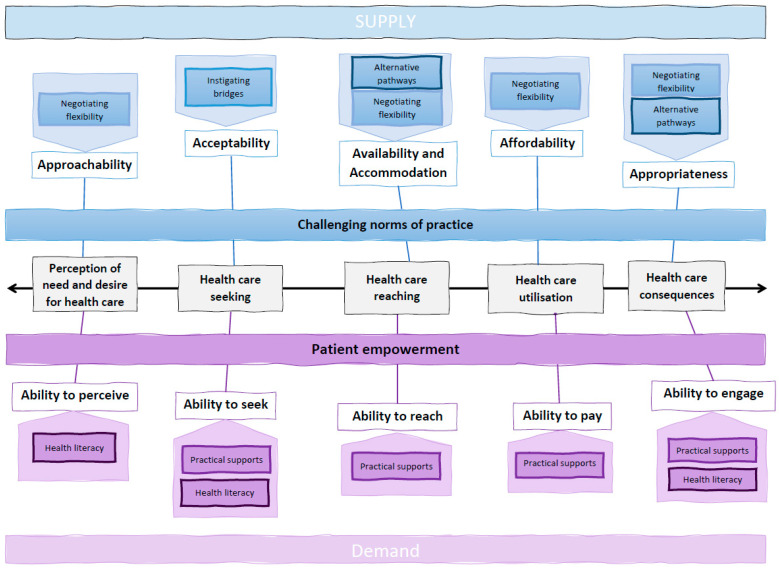
Early draft of thematic mapping of NN interview data over Levesque et al.’s [3] framework.

Reflexive thematic analysis was carried out in accordance with Braun and Clarke’s [34,35] six phases and was an ongoing and iterative process. Data immersion and familiarisation were a sense-making process through which initial understandings of the interviews were gained. This phase encompassed listening and re-listening to audio recordings and active reading and re-reading of transcripts [34,35]. In this phase, patterns across the data were identified and initial ideas noted [34,35]. Coding, the process of initial data organisation, was used to identify data (extracts) relevant to the research question and generate descriptive labels (codes) [34]. In this process, CHJ used NVivo 12 software to manage transcripts and coded data systematically. Transcripts were first deductively coded using Levesque et al.’s [3] conceptual framework. This was followed by a process of inductive coding to identify and organise data that fell outside Levesque et al.’s [3] framework. Codes were then organised around central thematic concepts [35]. CHJ, AM, and GM met regularly to discuss common threads between codes and how these related to the research question. In addition to enhancing rigour, this “crystallisation” process of engaging with differing perspectives and expertise when constructing codes added to the richness and depth of the data analysis [35,36,37]. Mind maps were also developed to assist in code sorting and to explore relationships between codes visually to identify potential themes and subthemes, which were then refined, collated, and cross-referenced against the entire data set (see Figure A2) [34]. Braun and Clarke [34] stress that theme presentation must go beyond description to clearly illustrate how each theme fits within the broader narrative and to support each theme with sufficient evidence. Accordingly, the findings are presented with illustrative extracts, with participants represented by pseudonyms [34].

### 2.3. Data Triangulation

The steps undertaken for data triangulation across the three stakeholder groups were adapted from the protocol developed by Farmer et al. [38]. The objective was to synthesise data from across the interviews to explore areas of “convergence, complementarity, and dissonance” [38]. The first step consisted of sorting the findings into areas of interest as they related to the research question [38]. The next step involved convergence coding, where commonalities across the findings related to NNs’ work were identified [38]. For example, “building relationships” was identified early in the data analysis of NN interviews. CHJ mapped this preliminary finding across data from NN patient and care professional interviews and explored whether there was agreement (i.e., the theme “building relationships” was wholly represented across the data, with consistent meaning and interpretation), partial agreement (i.e., the theme was not prominent across the data, with differences in meaning and interpretation), disagreement (i.e., there was conflicting data), or silence (i.e., the theme not represented across the other data) [38]. In this process, nuances in participants’ perspectives across the data were noted. Illustrative cases and supporting extracts reflective of each triangulated theme were also highlighted and then compared with each other and mapped across the Levesque et al. [3] framework, particularly where they sat with respect to the supply and demand sides and interface of access to health care [38]. Similar to Farmer et al.’s [38] description of a completeness comparison, the unique contribution of different data was considered in relation to the core themes and subthemes. For example, understandings of what was valuable about NNs’ work in “building relationships” differed slightly across the stakeholders, so these nuances were highlighted to provide additional richness and insight. As with the other analysis processes, data triangulation was iterative, and triangulated themes were continually revisited and discussed in detail by the research team.

## 3. Findings

The interviews provided clarity about the interface of access, where multidimensional (in)abilities and supply-side dimensions dynamically converge and diverge to enable or inhibit access. At this interface, NNs leveraged their experience, expertise, and independent position outside the silos of hospital departments and units to negotiate systems and services in partnership with patients and care professionals. Navigation work was driven by NN’s ability to identify and address individuals’ unique and intersecting care needs, which were usually multidimensional, fluid, and embedded in health, social context, and human experience.

*… it’s not only about [the patient’s] medical needs, it’s their psychosocial, it’s their physical, it’s a whole range of needs that probably are never going to be completely solved.*

NN Rita

NNs’ role was as a “bridge in the middle” (NN Margaret) at the “interface between them [patients] and the hospital” (NN Rita) or “any other service providers” (NN Lesley). Ellen, a hospital outpatient AHP, reflected that NNs needed to be “… people that can sit with complexity and think it through and not just push the panic button or narrow their role down”.

**Figure 1:**
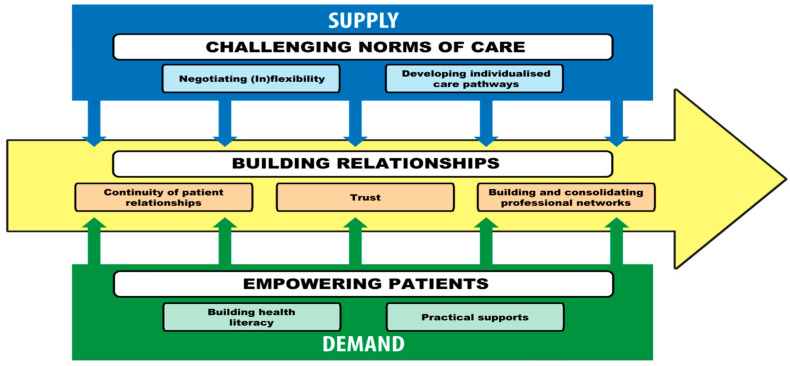
Study findings mapped across Levesque et al.’s [3] conceptual framework.

NNs’ work was highly contextualised to their specialty area, patient, and the setting in which care was delivered. Reflecting the complexity of practice, three key themes relating to how NNs operated at the interface of access were identified: (1) relationship building, (2) challenging norms of care, and (3) empowering patients. Building relationships was a cross-cutting theme that covered both supply and demand sides of access. Challenging norms of care sat most prominently on the supply side, whereas patient empowerment featured on the demand side (see Figure 1).

The following examination of findings explores these themes and associated subthemes, but to enhance understanding of the complex health care context in which NNs operated, we begin by briefly examining Henry’s experiences. As an NN patient, Henry’s narrative shows how experiences of accessing health care are determined by (in)abilities of access situated within his health and social context, juxtaposed with health system dimensions. Henry’s experiences reflect the multidimensional nature of access to health care and the (many) challenges encountered.

### 3.1. Henry

When we interviewed Henry in late 2022, he was in his 50s. Born in rural Queensland, he had limited schooling and could not read or write. Henry had been highly mobile throughout his life, and at the time of the interview lived on a pension in temporary accommodation in inner regional Queensland. Henry had a limited support network, no close family (his children were estranged), but some friendly neighbours. A close family member had passed away in tragic circumstances, and he experienced ongoing trauma associated with the loss.

Henry had multiple complex health needs. He had injuries sustained in an industrial incident, which left him unable to work, in constant pain, and reliant on walking aids. He had multiple chronic conditions, including emphysema, cardiovascular disease, and coeliac disease. Henry’s limited income meant he had to tightly budget all his expenses, including his medical expenses. When we spoke to him on a Friday, he had AUD 14 to last until the following Tuesday. His rent had recently increased, but a lack of alternative affordable housing options meant he could not relocate. Henry described many interrelated challenges that stemmed from his limited income, social isolation, education, and health conditions.

Henry recalled many interactions with the health system, including frequent hospital stays and negative experiences, which left him feeling isolated, overlooked, and stigmatised. He felt health care professionals were dismissive of his pain and health needs. His limited literacy meant following a gluten-free diet was extremely difficult as he struggled to navigate food labels, and outpatient appointments were frequently missed due to services communicating via letters and texts that he could not read; his file was repeatedly labelled “failure to attend”, and he was removed from waitlists. Behaviour change advice from clinicians was challenging and unrealistic because it did not account for his physical abilities, limited literacy, and social circumstances. Like many participants, Henry struggled to find a local GP he trusted and could afford. He had recently found a new general practice, but it was an hour’s drive away, adding further costs to his restricted budget. Henry’s new GPs referred him to the nurse navigation program.

### 3.2. Building Relationships

Relationships are central to health and social care, but limited opportunities for relationship building were a critical issue for NN patients, like Henry, and care professionals. Building relationships was the keystone of nurse navigation and central to their work supporting access to care. NNs facilitated multidimensional relationships with patients, families, and caregivers. They also established relationships with and between care professionals, with broader services and other NNs. Analysis of this theme identified three subthemes related to NNs’ work to foster relationships to facilitate access: (1) continuity of patient relationships, (2) trust, and (3) building and consolidating professional networks.

#### 3.2.1. Continuity of Patient Relationships

Feeling lost, disempowered, marginalised, and not having anyone to provide continuity or coordinate care were key barriers highlighted by many patients, including Henry. Acknowledging these shortfalls, NNs described their efforts to build genuine relationships with patients to foster a sense of ongoing connection. Many patients described NNs as approachable, a source of ongoing support, and a key reference point when care needs arose. For Henry, his experiences of being overlooked were reversed by the NN who “[was] there virtually holding my hand all the way through” (Henry).

NNs acknowledged that health services were not readily visible or welcoming to patients, which undermined their approachability. The NNs and care professionals interviewed recognised the impersonal nature of care provision and limited opportunities to connect with patients and understand their social context. Five-minute medicine “in and out… see[ing] a different person every time” (NN Therese) was described by NNs as a critical barrier, but they worked to become a key point of contact for patients.

Critically, NNs cared about patient participants’ lives, beyond matters of health. Angelina, a woman in her 70s, was living in a rural area, navigating cancer treatment and surgery, while caring for a family member with declining health. Angelina lived over an hour away from health care services and had a limited support network. Angelina’s NN noticed aspects of life that were important to Angelina and took time to get to know her beyond diagnosis and treatment.

*[NN] has been a marvel… sometimes we’re on the phone and I tell her what I’m cooking, talk about the kids. … But if I’ve got a problem, I can ring her. She’s apologised about being off for a day … I’ve never met anyone like her.*

Angelina, NN patient

Many patients worried about being a burden or getting a negative reaction from health care providers, particularly when they asked questions. Angelina expressed that, unlike past health care experiences, her NN was always approachable and available.

*She never gets irritated. She never gets cranky. She’s just amazing to me. I think sometimes, “Oh, you know, I’m going to annoy her”… [But] She’s always pleased to hear from me. She said, “If you weren’t going to ring me I was going to ring you”.*

Angelina, NN patient

#### 3.2.2. Trust

A lack of trust, embedded in experiences of alienation and marginalisation in the health system, as Henry’s case illustrated, marred some patients’ ongoing perceptions and expectations of future care. By investing in relationships, NNs became trusted points of connection and gained deeper insight into patients’ experiences and care needs. Building trust actualised patients’ perceptions of need and their ability to seek and (re)engage in care. For example, NN Rita recalled a patient who, as a former health professional with a high level of health literacy, was intensely distrustful and disengaged from care.

*… it didn’t matter how much health literacy she had because she was in such a dark place that—at the start, it was just about relationship building with her and delivering on your promises. Once we had that rapport, then she appeared to feel more comfortable in telling me things.*

NN Rita

Similarly, Kathleen, who had experienced multiple limb amputations, wanted to be seen as a person first and foremost. Her NN was one of the few health care professionals she trusted.

*…she’s watching everything that happened. Yeah, she’s my shining light … She’s physically, emotionally, everything, just hands on. … even though she didn’t have to and she still doesn’t have to, she is still doing it, so it’s a win/win for me.*

Kathleen, NN patient

Patient participants described NNs as their “champion”. This facet of the NN role was particularly important for people who had previous negative experiences and lost faith in the health system. For Julie, their NN was an advocate on her family’s behalf in interactions with the hospital and took all the angst out of her daughter’s hospital admission.

*… I could just be relaxed and not have to worry and knowing that if someone gave me grief, I just had to call [NN] and she was going to sort it out. I didn’t have to be the one, that would do it… [the NN] was there on my side and if I needed somebody, she would come at the drop of a hat. … I wasn’t there alone, if I didn’t want to be.*

Julie, caregiver for Gwen—a NN patient

Not all care professionals have the time or capacity to understand patients’ social context, but by investing in relationships and building trust with patients, NNs gained insight into people’s needs and were then able to feed back to broader care teams, thereby supporting the provision of appropriate care.

#### 3.2.3. Building and Consolidating Professional Networks

Fragmentation, particularly between hospitals, primary health care, and community social and welfare services, and limited collaborative relationships among professionals, challenged care professionals’ capacity to coordinate and integrate care for people with complex care needs. Seeking to redress this and strengthen opportunities for access, NNs fostered relationships with and between care professionals (including GPs, AHPs, medical specialists, and nurse practitioners) and across care settings and systems (including health, welfare, and community). These relationships were critical for building referral pathways and breaking down silos. NNs facilitated introductions between care professionals and kept them on the same page. NNs were excellent networkers, “linking me in with the right people” (Vivian, CSP) and facilitating connections between hospital and community services.

*I call [NN] my bridge because when it comes back to navigating hospital from an outsider’s perspective, that’s not necessarily easy. So [nurse navigation is] very much involved with establish[ing] relationships so I know who I can turn to if I need assistance.*

Jocelyn, CSP

NNs took a holistic and collaborative approach when building networks to ensure people’s needs were met. Vivian, a CSP, recalled a case in which an NN had coordinated multiple stakeholders to establish communication and care planning processes in person to support a person who was “getting constantly lost in the system”. NN Margaret clarified that navigation work did not replace existing care professional roles but supplemented and streamlined the whole patient care journey.

*So we don’t replace what anyone else does, we’re almost like a coach, so we sit above and kind of get to see how everything interacts across all areas of health and life. … we have all of these wonderful types of knowledge about how we can help streamline it.*

NN Margaret

Care professionals valued NNs’ ability to draw on their insider knowledge of the health system to forge connections across hospital, primary health care, and community settings, which made a significant difference to patient care. For example, Matilda, a hospital-based outpatient AHP, often drew on the NNs’ understanding of community resources to ensure appropriate supports were connected into the care of patients.

*… it gets overwhelming at times understanding what’s out there, how they all work, what the eligibility is, who has got capacity to take people on. So I would often work with the navigator … and say, you know, “I’m concerned about this person, I feel like they need X, Y and Z”. And then problem solve in conjunction with them.*

Matilda, hospital outpatient AHP

NN Caitlin noted hospital departments focused on specific aspects of the individual and lost sight of broader care goals, leading to poor outcomes. NNs and care professionals noted that building relationships with each other took time and was not always an easy process. Relationships required continuous investment but ultimately laid the foundation for better patient experiences and system improvements.

*… I’ve made a really big effort to build that rapport with the teams that I work with. I’m hoping that once you’ve done the groundwork … and you need to keep chipping away to make sure that they’re working, but I do find that it gets easier… I can see the improvements in the system.*

NN Caitlin

NNs also built relationships with each other to establish a strong community of practice. Through their community of practice, they were able to draw on each other’s expertise and specialised knowledge to enhance patient outcomes.

### 3.3. Challenging Norms of Care

Lack of fit at the interface of access was a significant barrier for care professionals supporting people with complex needs. Lack of fit was born out of the dominance of systems and their prescriptive approaches that overlooked complexity and problematised patients’ (in)abilities. NNs straddled the middle ground between care professionals, inflexible systems, and patients. They used their unique position and insight to challenge norms of care, “making health care equitable, because it’s not equitable, you fit in a box and if you don’t fit in that box then you’re a problem” (NN Margaret). Health care services prioritised acute episodes of medical care and viewed conditions in isolation, but NNs added context to challenge services and care professionals to see patients more holistically. Two key subthemes related to how NNs challenged norms of access included (1) negotiating (in)flexibility and (2) developing individualised care pathways.

#### 3.3.1. Negotiating (In)Flexibility

Prescriptive approaches to care provision place the onus of navigating barriers on individuals. NNs challenged norms to make systems work for patients. NNs promoted adaptability and inclusivity in health system and provider processes, particularly those related to approachability, accommodation, and affordability dimensions of care. For example, NN Caitlin described advocating for a service to provide alternative arrangements to accommodate a patient’s unique needs.

*We had one woman who couldn’t come on a Thursday and she was told, “You’ve got diabetes. The diabetes clinic is on Thursday.” That’s it. It was like, “That’s not it because we want her to come for care. We want her to engage.” … there’s always ways we can get around stuff.*

NN Caitlin

NNs advocated for personalised care experiences, sensitive to people’s needs and considerate of social context. They negotiated with providers to meet patients where they were, not where the system prescribed them to be. Advocacy work included ensuring patients could see the same clinician for outpatient appointments, scheduling multiple appointments for the same day to reduce hospital visits, and ensuring patients’ care teams were cognisant of patients’ care goals. NNs also supported service changes through involvement in service reviews that promoted shifts in workforce culture and symposiums, driving education to improve best practice.

NNs and care professionals all acknowledged system rigidity and lack of accommodations exacerbated barriers to care and contributed to experiences of alienation. Henry’s inability to read or write made managing and attending appointments difficult. Henry’s GPs had advised the hospital of his illiteracy, but the service kept sending him letters; consequently, he missed appointments and was removed from waitlists.

*… my GP got a letter from the hospital to say that I refused to attend, and then … [GP1] sent a letter back to them to say that he can’t read and write, I’ve told them all in the hospital, I don’t lie about it, and… then they turn around and say that he refused to attend. Well I didn’t refuse to attend because I didn’t know what it was for and what the letter was about.*

Henry, NN patient

Henry’s NN helped re-organise appointments and advocated with services to get him reprioritised. She also organised access to a text-to-voice phone application to enhance his access to information.

Prioritising treatment of acute presentations was a significant challenge for care professionals who were unaware of people’s social context. NNs, aware of this challenge, leveraged “knowledge about the client and the situation” (Hannah, CSP) and assisted care professionals to see the barriers patients were facing, contextualising people’s needs and (in)abilities to access care. Care professional participants valued this broad perspective, particularly NNs’ ability to look across different facets of people’s lives to identify potential challenges and ways to troubleshoot: “… [NNs] don’t have tunnel vision, they have a much more holistic approach” (Jocelyn, CSP).

Explanation of context also occurred on the patient side. NNs translated system processes for patients and worked as translators to ensure patients and health care professionals could understand where each was coming from. For example, NN Rita explained the hospital wait list processes to a patient, while also advocating with care professionals to reprioritise them.

*I try to sort of float in the middle. When somebody is saying, “Oh, I can’t understand why I have to wait this long for an appointment …”, then I explain to them about triaging … and try and be that voice of reason for people. But then I also try to advocate within the health service and will say to consultants, “Look, this patient has done all this work. They’re really committed and keen. I’d hate for us to lose this opportunity. Is there any way we could slot them into a cancellation or something?” to get them seen and to build on that keenness that they have …*

NN Rita

#### 3.3.2. Developing Individualised Care Pathways

Fragmentation, particularly the lack of coordination and continuity of care, was a critical impediment to access. In systems that struggled to accommodate multimorbidity, many people must “…jump from service to service and unless they’re savvy or lucky, they fall through” (NN Margaret). NNs worked with care professionals to develop tailored pathways to ensure timely access to the right services across systems (i.e., health, welfare, and community). Through connections that responded to individual circumstances, NNs reduced instances of inappropriate care.

*There’s these hundreds of services out there and if the patient needs 20, we’ll bring them in as opposed to having all the hundred there that they don’t really need because that’s just confusing. Or they actually don’t need 10, they need 15. So we’ll just see what the patient needs and do as best we can to link them in.*

NN Therese

Care professionals and patients valued NNs’ capacity to look across multiple services to identify what was needed and what was unnecessary. As Vivian, a CSP, explained, NNs brought the right people together.

Standardised care pathways often do not consider barriers caused by social context. When patients could not physically reach services, or were linked to inappropriate care, NNs coordinated alternative routes. For example, NNs had insight into processes and resource availability and worked to circumvent waiting lists to link patients with needed supports. Hannah, a CSP, noted

*…[NNs] are on the ground, they are in contact with service providers. So often they are more aware of what services are available. Then they feed back to us and tell us, “oh, such and such organisation has these services, their books are open for this”.*

Hannah, CSP

NNs’ extensive professional networks, which spanned systems and settings, were important for care professionals with limited opportunities to understand others’ roles and operating processes.

*…the most effective thing that [NN] has done … is educating both the staff at the hospital and educating us [in social community services] on systems and the processes and how things work. Because it is a bit of a minefield when you’ve never worked in either sector.*

Vivian, CSP

Aligning with the Appropriateness of Care dimension [3], NNs gained insight that could be used to initiate and consolidate changes within the system. Building and consolidating system changes involved demonstrating and codifying improvements in integrated care within and between services. NNs then worked with key stakeholders to disseminate the knowledge acquired and support incorporation into policy and practice.

### 3.4. Patient Empowerment

Patient empowerment was a core facet of the NN’s role in facilitating access to health care. Through efforts to empower people, NNs strengthened individuals’ abilities to perceive, seek, reach, pay, and engage in health care. Critically, patient empowerment activities were supported by NNs’ understanding of people’s (in)abilities and multidimensional care needs and their knowledge of dimensions of access to health care. Analysis of this theme identified two key subthemes: (1) building health literacy and (2) practical supports.

#### 3.4.1. Building Health Literacy

Through spending time with patients and developing deeper understandings of health practices, NNs recognised strengths and limitations in their patients’ health literacy, along with services’ incorrect assumptions about patients’ literacy. For Colin (NN patient) and Michelle (Colin’s caregiver), their NN was a vital connection to appropriate services and supports that would support Colin’s ongoing care.

*[NN]’s just pointed us all in the right direction[s] and been just so helpful with even organising [specialist health] appointments and for people to come out. … she just knows all the right people and the paths to take. … You just don’t know the steps to take and who to call and the processes. Without [NN]’s guidance, I would have been left behind.*

Michelle, caregiver for Colin—a NN patient

Limited literacy hindered people’s ability to perceive the need and desire for care and often stopped them from seeking and engaging in care, as Henry’s case clearly illustrates. NNs supported patients to engage in the management of health conditions in ways that recognised their level of understanding and context. NNs and care professionals acknowledged that limited health literacy meant some patients struggled to self-manage conditions. NN Lesley spent time working with patients to develop a “sick day plan” and thereby avoid frequent visits to the emergency department.

*… So we sort of try and equip them with self-management strategies so that when they are home they don’t panic. … So they’ve got, like, a sick day plan to say, okay, if this happens then I can do this. Instead of them presenting to [emergency department] for that minor thing, they know what to do at home and then they can resolve that issue.*

NN Lesley

Patients often lacked confidence to speak in clinical consultations and could be embarrassed and/or reluctant to seek support. To support literacy and build confidence, NNs adjusted communication strategies to meet people’s level of understanding. For example, using “basic blunt words” (NN Margaret) when talking about conditions and treatment plans, having frank discussions with care teams to ensure consistent information was shared, and supporting patients to feel comfortable speaking up and asking questions. NNs both advocated on patients’ behalf and coached them to advocate for themselves. To this end, the NNs supported people to participate in care decisions and prioritise their own goals when interacting with clinicians.

*There’s still a lot of stigma that doctors are these really intelligent busy people because they project that they’re busy. So patients often don’t want to inconvenience them, but I think our job is also to remind them that this is your health. This is your moment to be inconvenient, ask questions, be annoying, it’s your life.*

NN Margaret

#### 3.4.2. Practical Supports

Directing people to practical supports that assist with day-to-day living and support their abilities to reach, pay, and (re)engage in care was critical for enhancing access. Practical supports included the tools, materials, and resources that were often overlooked or considered outside the remit of health care. For example, Henry’s NN identified an App he could use when grocery shopping to determine if a product met his dietary requirements. This information significantly reduced his acute illness episodes and hospital admissions.

Social support, social isolation, housing instability, transport, and experiences of financial precarity were all noted as key barriers to care. NNs liaised with government and community services to ensure people were accessing the welfare supports they were entitled to (e.g., health care cards) and practical assistance that responded to multidimensional needs.

*I did have a young man a little while ago who was meant to be coming from [rural town] who had to go … [to public hospital in Brisbane] every day, literally just for a five-minute x-ray. He had no money. He wasn’t even registered for the [welfare payment]. He had nothing. But the [community centre] actually arranged somebody who could drive him in at 6:30 am every morning into the [hospital] and back.*

NN Therese

The (in)ability to pay and (un)affordability of health care were critical barriers to access that care professionals often had limited capacity to address, but NNs used their networks to find affordable alternatives, including subsidy programs, care plans, and cheaper medications, as well as assist patients in negotiating co-payments for services. In the case of a person who was a New Zealand citizen and unemployed due to a physically debilitating illness, NN Rita described activating reciprocal (between-country) health care agreements to ensure they were on the right benefit and could receive funding. Both NNs and care professionals emphasised that limited funds and onerous scheme conditions could further restrict patients’ ability to pay for and engage in care.

NNs work to empower people’s ability to engage in care frequently involved enhancing living environments. For example, NN Therese recounted arriving at a person’s house to find physical barriers that prevented them from engaging in post-operative care, living circumstances that would have otherwise been overlooked and undisclosed. She liaised with welfare and community organisations to improve the person’s living conditions, including coordinating practical modifications to the home.

*Somebody from Homecare Assist has gone through and put some rails in his bathroom. He had a little step up from the bathroom up to his bedroom and they’ve got a little ramp there now. He’s got a rail in the kitchen as well so when he sits on his stool, that helps him pull up. A gentleman from the church has come through and put a board underneath … his little couch so that he’s at a good height to get out of now and he’s also going to try and rig something up for his bed to help him in and out.*

NN Therese

## 4. Discussion

Drawing on the experiences of key stakeholders, this study explored how NNs facilitate access to health care for people experiencing complex needs. NNs acted as intermediaries at the interface between complex systems, care professionals, and individuals to negotiate access to health care. NNs demonstrated flexible practices, focused on the multidimensional care needs and abilities of individuals and adapted to the challenges experienced by patients and care professionals. Broadly, they facilitated access to health care by building relationships, challenging norms of care, and empowering patients.

Despite well-meaning person-centredness rhetoric, navigating issues of access tends to be considered an individual responsibility, while the health system’s responsibility in supporting and addressing needs is often overlooked [6,39]. The experiences of patient participants highlighted that the health care system is often unable to see and respond to multidimensional care needs, which further confounds (in)abilities of access and contributes to poor health outcomes. These findings are consistent with broader research, which continues to describe the health care systems’ inability to recognise and respond to complex care needs, thereby potentially contributing to patient harm [1,40,41]. NNs acknowledged that patients were not passive health care travellers, but nonetheless, the health system often made uncontextualised assumptions about care needs and abilities. By negotiating greater flexibility in care arrangements, developing individualised care pathways, and building system connections, NNs demonstrated to health services how they could respond to people’s complex social lives and change the experiences of intersecting health inequities to improve access to care.

As demonstrated in previous studies, NNs took a “global view” of the people they supported, seeing patients as people and, in so doing, facilitating experiences of access that were more relational and responsive to the complexities of everyday lives [25,42,43]. Olthuis et al. [44] observed that “better understanding of patients’ lived experiences enabled healthcare professionals to attune to what individual patients deemed important in their lives”. NN work recognised that patients were “creative agents” who acted to shape the world around them and the “structures of power and control within which they [were] embedded” [45]. Moving away from paternalistic notions of adherence, NNs’ work aligned with the concept of concordance—care driven by mutual understanding, partnership, and collaboration [46,47].

The commodification of health care interactions, influenced by increasing service demand and operational pressures, limits opportunities for care professionals to investigate social context and understand the complexity of people’s experiences, and this has resulted in the loss of continuity and depth of patient–provider relationships [40,47,48,49]. Building relationships with patients, families, and caregivers, and with and between care professionals, was central to NNs’ work and underpinned their ability to mediate access to care. This finding reminds us of how critical relationships are in health care, particularly as mutual understanding, commitment, and trust are essential when navigating complex health and social care [1,48,50,51,52]. Building relationships and (re)establishing trust is critical to (re)engage people with the health system, but it is also essential for ensuring practitioners genuinely engage with their patients [20,21]. Voorhees et al. [13] argue that (positive) interactions between people with care needs and the health care workforce determine access. Relational continuity is essential for ensuring that these interactions allow clinicians to build trust with their patients and have “comprehensive knowledge of the patient as a whole person” [51]. The current study clearly illustrates how relational continuity strengthens access to health care and highlights how NNs are uniquely positioned, as agents working both within and outside the health system, to negotiate access to health care through their relationships with patients and care professionals.

Access to health care begins with perception of need—as people’s health and social needs change, so too must the system’s responses to these needs. The findings suggest many gaps at the interface of access related to needs, and from whose perspective these needs are perceived and how they are responded to (or not). What matters most and the capacity to respond differ across people with care needs, care professionals, and systems; misunderstandings contribute to poor outcomes on all fronts [53]. Patients and health services are constantly defining and redefining what warrants their attention through dynamic and continuous negotiating processes [54]. This study further demonstrates that we must move beyond an isolated focus on individual characteristics and acknowledge the role systems play in perpetuating and reinforcing barriers to care and inequities of access [52,55,56]. While equity is framed as a critical component of health care performance, narrow lenses, particularly those focused exclusively on individual responsibilities, assume a suite of abilities that some people may simply not have access to. This misguided focus on individual abilities misses opportunities to assess health care structures and determine how dimensions of access to health care must work to create and maintain equitable outcomes [55,56]. Genuinely applied person-centred approaches—those that look beyond “health” to consider social context—must be prioritised to dismantle barriers and respond to people’s multidimensional care needs [55]. Navigation models are one route to understand the needs of patients, care professionals, and systems, and they offer insights that, if leveraged, provide an opportunity to reform systems that are no longer fit for purpose.

Levesque et al.’s [3] framework was developed to align with notions of person-centredness and is focused on access to health care. Continuing the evolutionary progression of access theory, the findings suggest the framework should be expanded to account for notions of integrated care (across systems and settings). The continued siloing of understandings of access to care (i.e., focusing on “health” without consideration of “social” care) limits the ability to consider the multidimensionality of care needs, which are not bound within discrete care systems. The challenges described by care professionals and NNs’ work overcoming them, demonstrated the need to bridge the many gaps between health and social care to account for experiences of access holistically. The findings, therefore, highlight a need to develop a model of access to health and social care, or more simply, of access to care (where “care” is a catch-all). To strengthen integration activities in practice, understandings of access must continue to evolve to reflect contemporary aspirations of care systems.

This research focused on one nurse navigation model and provides a highly contextual account, triangulating insights from NNs, people with complex care needs, and care professionals. Other models of nurse navigation have been developed and implemented elsewhere to meet their local priorities [57]. The findings are not intended to be reflective of the operation of other nurse navigation models. This was reflexive qualitative research, which included a strong component of co-design with the NN team. Co-design processes are increasingly recognised as the gold standard for research embedded within the community and concerned with experiences of social marginalisation [32]. However, co-design processes were limited to the NN team. Expanded co-design processes to include other care professionals and, most importantly, people with complex needs, would further strengthen the findings of this work by diversifying the perspectives involved.

This research provides rich insights from a small, highly heterogeneous cohort, but it did not consider the experiences of community groups who are known to experience added health and social barriers to care, including experiences of First Nations peoples or people from culturally and linguistically diverse communities. As understandings of navigation continue to be developed, the experiences of diverse population groups must be included. The potential of bias is a further limitation that warrants careful consideration. NNs led to the recruitment of NN patient participants and guided the recruitment of care professional participants. This recruitment strategy may have introduced selection and social desirability bias. Additionally, the care professionals who participated had varied interactions with the NN team. However, the objective of this research was to gain an understanding of NN practice, not to evaluate the service. Moreover, the triangulation of stakeholder perspectives was an important feature of this research, which provided multidimensional insight into the NN role and strengthened confidence in the findings.

## 5. Conclusions

This study presented an in-depth analysis of NN work to facilitate access to care, drawn from the experiences of key stakeholder groups. The findings illustrate how NNs facilitated access for people with complex care needs and supported care professionals by bridging the many obstacles encountered at the interface of systems and individuals. Specifically, this research highlighted the importance of holistic and relational approaches, embedded in identifying and responding to multidimensional care needs. Such insights are essential for our understanding of access to health care, but most particularly for those individuals who experience complex care needs and inequities of access.

## Data Availability

The original contributions presented in this study are included in the article. Further inquiries can be directed to the corresponding author.

## Supplementary Materials

The following supporting information can be downloaded at https://www.mdpi.com/article/10.3390/ijerph22111631/s1, Document S1: NN Interview schedule; Document S2: NN Patient interview schedule; Document S3: Care professional interview schedule; Document S4: Connection Diary Template.

## Abbreviations

The following abbreviations are used in this manuscript:

AHPs	allied health professionals
CSPs	community-based social and welfare professionals
GPs	general practitioners
NNs	nurse navigators
WMHHS	West Moreton Health and Hospital Service
