# Supplementary material for: “Float[ing] in the Middle” Nurse Navigators and the Interface of Access to Care
Source: Int J Environ Res Public Health. 2025 Oct 26;22(11):1631. doi: 10.3390/ijerph22111631 (PMC12651983; doi:10.3390/ijerph22111631)
Supplement: Supplementary file 1 [file ijerph-22-01631-s001.zip › ijerph-3903091-supplementary.pdf]

Supplementary Materials

"float[ing] in the middle" Nurse navigators and the interface of access to care

Document S1: NN Interview schedule

Research Questions:

Do NNs facilitate access to care for people with medically and socially complex care needs?

How do NNs work with HCPs to facilitate access to care?

How do NNs work with patients to facilitate access to care?

Aim: Explore role of NNs in facilitating patient-centred access to quality care (explore the black box of practice)

**Final Question**	**Prompts**
**Broadly, how do you conceptualise access to care? – what does access to care mean for you?**<br>**Do you see the NN role as at the interface of access, between patients, care systems and care providers?**<br>**What are some of the common barriers to care your patients face?**<br>**What are some of the barriers that care providers that you work with face?**	**Understanding patient social context.**<br>• **psycho-social barriers patients experience?**
**Do you think health services are approachable for your patients? (explain)**<br>**Do patients understand when they need to access services?**<br>**What do you do to bridge that gap?**	**Perception of need**<br>• health literacy<br>• health beliefs<br>[if have own advocate]<br>[self-management]<br>**Approachability**<br>• transparency<br>• outreach<br>• Information<br>• screening<br>**Connections to primary care**<br>**Systems initiatives**

**What are some of the financial barriers your patients face?**<br>**Beyond Medicare and the PBS, what are services doing to address these?**<br>**What do you do to bridge the gap?**	**Ability to pay**<br>• income<br>• social capital<br>• insurance coverage<br><br>**Affordability**<br>• direct and indirect costs<br>o impact on work / loss of income<br>• opportunity costs
**Do you think your patients are able to actively engage in their care?**<br>**How do you support patients to actively engage in care?**<br>**How do you work with HCPs to ensure patients are receiving appropriate care?**	**How facilitating trust?**<br>**Links to primary care**<br>Understanding patient goals for care<br>**Tackling with systems initiatives**<br><br>**Ability to engage**<br>• empowerment<br>• health literacy<br>• adherence<br>• caregiver support<br><br>**Appropriateness**<br>• interpersonal quality<br>• coordination and continuity<br>**[being proactive vs reactive]**<br>**[links to community]**<br>**[broader scope to what can do in system]**
**What are some of the challenges you encounter in your role, (as you negotiate the interface of access)?**	**What enables you to negotiate between making sure the person is receiving the care that they want/need but also that you are meeting system criteria?**<br>[tick box/checklists]<br>[multidisciplinary team dysfunction – organisational barriers – staff turn-over, political]

**How do services account for values, beliefs and understandings of the community members being served?**<br><br>**Are patients confident to seek help when they need it?**<br><br>**How do you work to try to address these issues?**	**Ability to seek**<br>• Personal<br>• social values<br>• culture<br>• gender<br>• autonomy<br><br>**Acceptability**<br>• professional values<br>• norms<br>• culture<br>• gender<br><br>**Connections to primary care**<br><br>**Systems initiatives**<br><br>We know patients often come into the nurse navigation service at points of crisis – how do NN put supports in place to support their patients to seek out care in the future?<br><br>Do services meet patient's diverse cultural needs?
**What physical barriers to access do your patients face?**<br><br>**How accommodating are services to addressing these needs?**<br><br>**How do you work to try to address these physical barriers?**	**Ability to reach - Personal mobility**<br>• living environments<br>• transport<br>• mobility<br>• social support<br><br>**Availability and accommodation**<br>• geographic location<br>• hours of operation<br>• accommodation<br>• appointment mechanisms (e.g. organisational flexibility able to negotiate with providers)<br><br>**Systems initiatives**

Document S2: NN Patient interview schedule

Queensland Government
The University Of Queensland
AUSTRALIA

Patient interview schedule

Interview length: up to 120 minutes

1. **Welcome and introduction**

- Thank for participating
- Explain purpose and importance of the study and their involvement
- Ethical issues - confidentiality etc.
- Reminder – participant may choose not to answer questions, take a break or end interview at any time.
- Discuss list of resources/support. Explain that you are not a professional counsellor and should participant want to get some support- we provided a list of resources
- Sign consent form
- Reimbursement of $50 gift card
- Check if participant request interview transcript
- Permission to start audio recording

2. **Demographics**
   1. Age ___________
   2. What is your gender? (you may choose more than one)
      a. Male
      b. Female
      c. Intersex
      d. Transgender ______________________
      e. I identify as: ______________________
   3. Postcode ___________
   4. Do you identify as Aboriginal or Torres Strait Islander?
      a. Aboriginal
      b. Torres Strait Islander
      c. Aboriginal and Torres Strait Islander
      d. Neither Aboriginal nor Torres Strait Islander
   5. Do you have a regular General Practitioner?
      a. Yes
      b. No
      c. Other: ______________________
   6. How long have you been with a nurse navigator?

Interview is a conversation – broad open-ended questions and focus in on key research themes

NURSE NAVIGATION PATIENT INTERVIEW SCHEDULE

1. **Tell us about you – where did you grow up etc, parents, jobs, school?**

2. **When growing up did you have many health issues/interactions with the health system?**

   - Did you go to the doctor for things?

3. **What's your health like now?**

   When did (first illness) first start? (talk us through that episode)

   - Who did you see / why them?
   - How did you know where to go? (easy to find?)
   - How do you go getting to services – transport, location, time, mobility?
   - Were there costs?
   - When you go to service x, do you feel comfortable asking questions? Seeking the information you need?
   - Was the care what you expected? Were there challenges / things that could have been done better?
   - Any carers? Multi health profs? Challenges?

4. **When did you first meet NN?**

   - How did you get put in touch with her?
   - How long have you been working with x?
   - What has x done/does to support you? (interactions with HP, educ., supports)
   - What's your relationship with x like? (easy to talk to, understanding?) Has it changed/developed?
   - What have they helped to manage? Challenges overcome?
   - Who has she linked you in to?
   - Anything be improved?

Document S3: Care professional interview schedule

Care Professional interview schedule

Interview length: up to 60 minutes

1. Welcome and introduction

- Thank for participating
- Explain purpose and importance of the study and their involvement
- Ethical issues - confidentiality etc.
- Reminder – participant may choose not to answer questions, take a break or end interview at any time.
- Discuss list of resources/support. Explain that you are not a professional counsellor and should participant want to get some support- we provided a list of resources
- Sign consent form
- Reimbursement of gift card if private practice general practitioner
- Check if participant request interview transcript
- Permission to start audio recording

2. Demographics

1. What is your gender? (you may choose more than one)
   a. Male
   b. Female
   c. Intersex
   d. Transgender ________________________
   e. I identify as: ________________________
2. Area/s of speciality ________________________
3. Length of time in practice ________________________

Research Questions:

Do NNs facilitate access to care for people with medically and socially complex care needs?

How do NNs work with HCPs to facilitate access to care?

How do NNs work with patients to facilitate access to care?

**Final Question**	**Prompts**
How would you describe your role?<br><br>Who are your patients?	Setting context<br>• Where do you work?<br>• Who do you work with?
Broadly, how do you conceptualise access to care? – what does access to care mean for you?	We see the NN role as at the interface of access, between patients, care systems and care providers
**What are some of the barriers that you face as GP – in practice**?<br>What are some of the common barriers to care your patients face?	• Barriers to access
When did you first become aware of nurse navigation /NN X?	- How did you get put in touch with x?<br>- How long have you been working with x?<br>• Talk through that/those interaction?<br>• **What is your ongoing relationship / interactions like?**<br>o Same or different nurse navigators?
What role do you see NN play in enhancing access to care?<br>- System side / patient side	How do you see NN influencing patient:<br>• engagement, availability, affordability, utilisation, approachability, appropriateness of services received by patients?<br>• How do you see NN as influencing patient ability to perceive, seek, reach, pay and engage in care?<br>• Can you give example?

Do / how do the nurse navigators support your role?	• What aspects of the NN role are helpful to you in your role?<br>• What are some of the challenges in working with a NN?<br>- What has x done/does to support you? (interactions with HP, educ., supports)<br>- What's your relationship with x like? Has it changed/developed?<br>- What have they helped to manage? Challenges overcome?<br>- Anything be improved?<br>How working with them to increase access -

Document S4: Connection Diary Template

Queensland Government

THE UNIVERSITY OF QUEENSLAND AUSTRALIA

Nurse navigator connection diary

**Title**	Enhancing access to quality care: the case of nurse navigation
**Short Title**	Nurse navigation and access to care
**Protocol Number**	HREC/2020/QWMS/67359
**Principal Investigator**	Ms Clare Hannan-Jones
**Co-investigator(s)**	Associate Professor Allyson Mutch<br>Emeritus Professor Geoffrey Mitchell

The purpose of this diary is to record who you are connecting with in the care of patients i.e. the different professionals/services you interact with in your navigator role.

The aim of this diary is not to follow any particular patient journey, but to record all contacts related to your navigator role over the course of 3-5 days (e.g. within the hospital, primary care, social system, financial services). Patient interactions are excluded from this diary.

Please assign your patient's pseudonyms (e.g. Gary becomes Patient A) and only include these pseudonyms in the diary.

Entries may include contacts not directly associated with any particular patient (see example 2). Where multiple people are included in the same interaction, please indicate so (see example 3).

This information is to be collected over 3-5 days (these do not need to be concurrent), and may be recorded in hard copy or digitally.

Example entries

1	17/8/2020	Patient A	Dr Skylar from Ipswich Family Practice	Discussed review of medications
2	17/8/2020	n/a	Clare from University of Queensland	Email correspondence on upcoming research
3	17/8/2020	Patient B	Mandy, Queensland Positive People<br>Jamila, Multicultural Queensland<br>Harry, Department of Human Services<br>Philippa, NDIS coordinator	Phone conference of patient case

If you have further questions, or for more information please contact:

Clare Hannan-Jones
PhD Candidate School of Public Health
The University of Queensland
E: clare.hannanjones@uqconnect.edu.au
P:

Associate Professor Allyson Mutch
Senior Lecturer School of Public Health
The University of Queensland
E: a.mutch@uq.edu.au
P: 07 3346 4682

Connection Diary

Date	Patient Identifier (e.g. patient A)	Contact (e.g. person B from organisation X)	Comment (e.g. spoke with social worker from X to arrange case conference)
